# Physical activity and risk of multiple sclerosis: A Mendelian randomization study

**DOI:** 10.3389/fimmu.2022.872126

**Published:** 2022-09-21

**Authors:** Chunyu Li, Junyu Lin, Tianmi Yang, Yi Xiao, Qirui Jiang, Huifang Shang

**Affiliations:** Department of Neurology, Laboratory of Neurodegenerative Disorders, National Clinical Research Center for Geriatrics, West China Hospital, Sichuan University, Chengdu, China

**Keywords:** physical activity, multiple sclerosis, Mendelian randomization, genetic correlation, causation

## Abstract

Multiple evidence from epidemiological studies has suggested association between physical activity and risk of multiple sclerosis (MS). However, the conclusion was still controversial between studies, and whether the association was causal or confounded is elusive. To evaluate the role of physical activity with different intensities in the risk of MS, we first estimated their genetic correlation, and then conducted two-sample and multivariable Mendelian randomization analyses based on summary statistics from previous large genome-wide association studies. A significant genetic correlation was identified between moderate physical activity and the risk of MS (genetic correlation: -0.15, SE=0.05, P=2.9E-03). Meanwhile, higher moderate physical activity was significantly associated with a reduced risk of MS (OR:0.87, 95% CI:0.80-0.96, P=3.45E-03). Such association was further verified using summary statistics from another study on overall physical activity (OR:0.36, 95% CI:0.17-0.76, P=6.82E-03). The results were robust under all sensitivity analyses. Current results suggested moderate physical activity could reduce the risk of MS. These findings help better understand the role of physical activity in MS, and provide some lifestyle recommendations for individuals susceptible to MS.

## Introduction

Multiple sclerosis (MS) is a devastating neurodegenerative disorder which affects the brain and spinal cord, slowly robbing patients of their physical mobility, vision and balance (1). Several factors could influence the susceptibility of MS, like genetic background and environmental factors such as smoking and vitamin D deficiency (2). Identifying risk factors for MS could help better understand the pathogenesis, and provide care and therapeutic strategies for patients and clinicians.

Physical activity can improve body health and has been suggested to be beneficial in several neurodegenerative disorders including MS. A previous case-control study comprising 628 men with MS and 6187 matched controls identified that the patients had lower physical working capacity in adolescence compared with controls (OR=0.94, 95% CI: 0.89-0.99, P=0.026) (3). Another study among 1904 MS cases and 3694 controls identified vigorous physical activity was inversely associated with the risk of MS (OR=0.74, 95% CI: 0.63-0.87, P<0.001), suggesting the potential protective role of physical activity against MS (4). Meanwhile, physical activity was shown to significantly reduce symptoms such as fatigue in patients with MS (5, 6). However, too aggressive physical activity might also bring on severe fatigue and too much stress in the muscles, which might be harmful to patients with MS. Additionally, a previous case-control study investigating lifestyle factors and risk of MS among 80 cases and 160 controls reported no significant difference in physical activity level between MS patients and controls (7). Another study in two prospective cohorts found that women in the highest physical activity quartile had a 27% reduced rate of MS (RR=0.73, 95% CI: 0.55-0.98, P=0.08), but the trend was not present in 6-year lagged analyses, suggesting the reduced physical activity might be in response to subclinical MS (8). And individuals with MS might even be more physically active than controls before diagnosis of MS in a previous case-control study of 200 newly diagnosed MS patients and 202 matched controls (OR=2.3, 95% CI:1.4-3.9) (9). Nevertheless, such observational studies might be biased by unavoidable confounding factors and a relatively small sample size. It could not be determined whether worsened motor symptoms in MS led to less physical activity or vice versa. Therefore, whether physical activity has a beneficial role in the susceptibility of MS is still elusive.

Genetic data has provided valuable insights into the causes and risk factors of complex diseases (10, 11). In the current study, we performed two-sample Mendelian randomization (MR) analysis to explore the causal role of physical activity in the risk of MS. With valid instrumental variables, the MR approach is less susceptible to reverse causation or confounding factors which may influence interpretations of results from conventional observational studies. As a result, we identified that moderate physical activity was causally associated with a reduced risk of MS.

## Methods

### Datasets

Exposure data were from a recent GWAS on physical activity among 377,234 participants from the UK Biobank (12). A touchscreen questionnaire was used to measure physical activity during work and leisure time. Four physical activity phenotypes from this study were analyzed, including self-reported moderate-to-vigorous physical activity (MVPA), self-reported vigorous physical activity (VPA), overall acceleration average (AccAve), and 2-3 days/week or more in doing strenuous sports or other exercises for a duration of 15-30 minutes or greater (SSOE) (**Supplementary Table 1**). As a replication, we further analyzed summary data from another GWAS on overall physical activity measured using wrist worn accelerometers (N=91,105) (13) (**Supplementary Table 1**). The overall activity levels were measured by average vector magnitude for each 30-s epoch, which was the recommended variable for activity analysis. Single nucleotide polymorphisms (SNP) with genome-wide significance (P<5E-08) from the original studies were clumped based on the 1,000 Genomes Project linkage disequilibrium (LD) structure. Index SNPs (R^2^<0.001 with any other associated SNP within 10 Mb) with the minimum P value were kept as instrumental variables. Furthermore, we used the PhenoScanner v2 tool to check for variants associated with other phenotypes (P<5E-08) which might affect the risk of MS independent of physical activity (14).

Summary statistics of outcome were from the largest GWAS on MS involving 47,429 cases and 68,374 controls of European descent (15). As a replication, we further analyzed summary statistics from another GWAS on MS (N_case_=14,802, N_control_=26,703) (16). Harmonization was undertaken to rule out strand mismatches and ensure alignment of SNP effect sizes.

### Genetic correlation

We estimated the genetic correlation between physical activity and MS using the LDSC and GNOVA methods with default parameters (17). The LDSC method quantifies the genetic correlation by exploiting the relationship between association test statistics and LD score expected under polygenicity. GNOVA calculates the genetic correlation based on genetic covariance and variant-based heritabilities, providing powerful statistical inferences that are robust to LD and sample overlap. All the SNPs in physical activity and MS together with reference data derived from the 1000 Genomes Project European population were utilized. A P value below 0.01 (0.05/5) was considered statistically significant after the Bonferroni correction.

### Mendelian randomization analysis

We hypothesized that physical activity as a protective factor could causally decrease the risk of MS, and the following assumptions were satisfied: the instrumental variables are associated with physical activity; the instrumental variables are not associated with confounders; the instrumental variables are associated with MS through physical activity (namely horizontal pleiotropy should not be present) (**Supplementary Figure 1**).

To evaluate the causative effect of physical activity on the risk of MS, we performed a two-sample MR analysis using the random effects inverse variance weighted (IVW) method, which is most widely used in MR studies and could provide robust causal estimates. A P value below 0.01 (0.05/5) was considered statistically significant after the Bonferroni correction. We further verified the results using the weighted median method, which generally has greater power with a positive causal effect. In addition, we conducted comprehensive sensitivity analyses to estimate potential violations of the model assumptions in the MR analysis. We conducted Mendelian randomization pleiotropy residual sum and outlier (MR-PRESSO) analysis and leave-one-out analysis to detect outlier instrumental variables. Outlier instrument variables identified by the MR-PRESSO analysis were removed step-by-step to reduce the effect of horizontal pleiotropy. Cochran’s Q test was executed to check heterogeneity across the individual causal effects. MR-Egger regression was performed to evaluate the pleiotropy of instrumental variables. Reverse causal inference with Steiger analysis was conducted to explore whether MS has a causal impact on physical activity. To evaluate the strength of each instrumental variable, we computed the F-statistic of each SNP. The statistical power was calculated using an online tool at http://cnsgenomics.com/shiny/mRnd/. The statistical analyses were conducted using R package TwoSampleMR 0.5.5.

## Results

**Figure 1:**
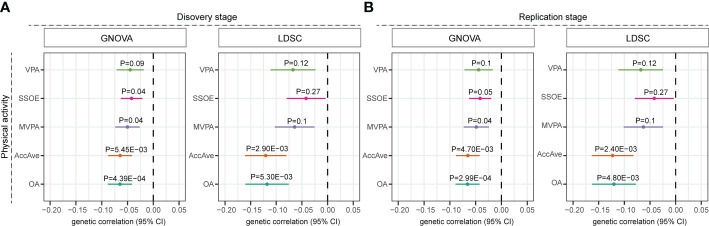
Genetic correlation between physical activity and multiple sclerosis. Genetic correlation was estimated using the GNOVA and LDSC methods in the **(A)** discovery and **(B)** replication stages. Error bars indicate 95% confidence intervals. AccAve, overall acceleration average; MVPA, self-reported moderate-to-vigorous physical activity; VPA, self-reported vigorous physical activity; SSOE, strenuous sports or other exercises; OA, overall activity.

We first estimated the genetic correlation between physical activity and the risk of MS. We detected a significant and negative genetic correlation between MS and AccAve (genetic correlation: -0.15, SE=0.05, P=2.9E-03), overall activity (genetic correlation: -0.12, SE=0.04, P=5.3E-03) using the LDSC method (**Figure 1A**). Such association was further verified in the replication stage for AccAve (genetic correlation: -0.12, SE=0.04, P=2.4E-03) and overall activity (genetic correlation: -0.12, SE=0.04, P=4.80E-03). Similar results were identified using the GNOVA method (**Figure 1B**).

**Figure 2:**
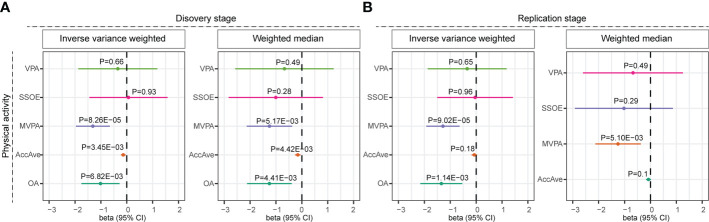
Forest plot showing results from the Mendelian randomization analysis. Mendelian randomization results were estimated using the inverse variance weighted and weighted median methods in the **(A)** discovery and **(B)** replication stages. Estimates are per 1 standard deviation increase in the trait. AccAve, overall acceleration average; MVPA, self-reported moderate-to-vigorous physical activity; VPA, self-reported vigorous physical activity; SSOE, strenuous sports or other exercises; OA, overall activity.

Then we analyzed the role of physical activity in the risk of MS using the two-sample MR approach. Results showed that each one standard deviation increase in AccAve (OR=0.87, 95% CI: 0.80-0.96, P=3.45E-03) and MVPA (OR=0.27, 95% CI: 0.14-0.52, P=8.26E-05) was significantly associated with a reduced risk of MS (**Figure 2A**, **Supplementary Figures 2, 3**). Meanwhile, higher overall activity was significantly associated with a reduced risk of MS using summary statistics from another GWAS on physical activity (OR=0.36, 95% CI: 0.17-0.76, P=6.82E-03) (**Figure 2A**). These associations were further verified using the weighted median method (**Figure 2A**). In the replication stage, higher MVPA (OR=0.28, 95% CI: 0.15-0.53, P=9.02E-05) and overall activity (OR=0.26, 95% CI: 0.12-0.59, P=1.14E-03) were significantly associated with reduced risk of MS (**Figure 2B**). In contrast, no association was identified between SSOE, VPA and MS, suggesting too strenuous physical activity might not be protective against the risk of MS (**Figure 2**).

Furthermore, we performed extensive sensitivity analyses to detect potential violations of the MR model assumptions. No heterogeneity of effects was detected by the Cochran’s Q test (**Table 1**). The F statistics of all the instrumental variables were above 10 (ranging from 29 to 82) (**Supplementary Table 2**), indicating valid strength of the instrumental variables. No apparent horizontal pleiotropy was observed as the intercept of MR-Egger was not significantly deviated from zero (**Table 1**). Meanwhile, no potential instrumental outlier was detected by the MR-PRESSO analysis. The Steiger test suggested correct directionality (**Table 1**). The leave-one-out results suggest that the causal effect was not driven by a single instrumental variable (**Supplementary Figures 2-6**). Given the type 1 error of 0.05, we had sufficient power (> 80%) to detect the association between MVPA, overall activity and MS (**Supplementary Table 3**).

**Table 1:** Heterogeneity and horizontal pleiotropy analyses between physical activity and multiple sclerosis.

stage	exposure trait	Heterogeneity test			Horizontal pleiotropy			MR-PRESSO P value	Steiger test	
		Q	Q df	P value	Egger intercept	SE	P		correct direction	P value		
discovery	AccAve	3.64	3	0.30	0.09	0.06	0.30	0.35	TRUE	5.86E-05
	MVPA	19.89	17	0.28	0.04	0.03	0.18	0.31	TRUE	4.62E-11
	VPA	3.67	5	0.60	-0.01	0.06	0.88	0.62	TRUE	2.00E-09
	SSOE	16.14	12	0.18	0.03	0.04	0.48	0.18	TRUE	5.50E-14
	OA	2.29	2	0.32	0.08	0.07	0.45	n.a.	TRUE	7.20E-15
replication	AccAve	11.59	5	0.05	-0.05	0.06	0.47	0.04	TRUE	1.81E-01
	MVPA	19.44	17	0.30	0.04	0.03	0.23	0.30	TRUE	4.21E-02
	VPA	3.68	5	0.60	-0.01	0.06	0.88	0.62	TRUE	1.65E-03
	SSOE	14.94	12	0.24	1.43E-04	0.03	1.00	0.26	TRUE	4.53E-04
	OA	0.06	1	0.81	n.a.	n.a.	n.a.	n.a.	TRUE	2.38E-06

Cochran’s Q statistic was used to detect heterogeneity about the inverse variance weighted estimate in the Mendelian randomization analysis. Q, Cochran’s Q test estimate; df, Cochran’s Q test degrees of freedom; SE, standard error; AccAve, overall acceleration average; PA, physical activity; MVPA, moderate-to-vigorous PA; VPA, vigorous PA; SSOE, strenuous sports or other exercises; OA, overall activity.

Lastly, we used the PhenoScanner tool to check if the SNPs used as instrumental variables in the MR analysis were associated with other phenotypes. Several SNPs were associated with body mass index (BMI) which was suggested to affect the risk of MS (18) (**Supplementary Table 4**). Therefore, we further performed multivariable MR to elucidate the causal relationship between physical activity and MS adjusting potential pleiotropy due to BMI. The summary data of BMI was obtained from GWAS published by the Genetic Investigation of Anthropometric Traits (GIANT) consortium (19). As a result, a nominal significant association was still identified between moderate physical activity and MS in the multivariable MR analyses adjusting for BMI (**Table 2**).

**Table 2:** Mendelian randomization estimates between physical activity and multiple sclerosis adjusting for body mass index.

stage	exposure trait	beta	SE	P value
discovery	AccAve	-0.11	0.05	0.017
	MVPA	-1.77	0.50	4.25E-04
	VPA	-1.19	1.39	0.389
	SSOE	0.49	1.02	0.630
	overall activity	-0.85	0.41	0.036
replication	AccAve	-0.10	0.05	0.030
	MVPA	-1.99	0.61	0.001
	VPA	-1.19	1.39	0.389
	SSOE	0.84	0.99	0.399
	overall activity	-0.85	0.41	0.036

AccAve, overall acceleration average; PA, physical activity; MVPA, moderate-to-vigorous PA; VPA, vigorous PA; SSOE, strenuous sports or other exercises.

## Discussion

Previous epidemiological studies have suggested physical activity was beneficial against susceptibility and symptoms of MS, but such a conclusion was still controversial across studies. Meanwhile, unmeasured confounding factors in clinical studies could potentially bias the association evidence. Two previous MR studies analyzed the role of physical activity in the risk of MS (20, 21), but got different results. However, the sample sizes in these two studies were much smaller, and only single type of physical activity was analyzed without adjusting for potential pleiotropy from other traits. In the current study, we systematically investigated the causative role of physical activity with different intensities in the risk of MS using the genetic correlation, two-sample and multivariable MR approaches. We identified a significant negative genetic correlation between physical activity and MS. Meanwhile, moderate physical activity was causally associated with a lower risk of MS. These findings provided a better understanding of the role of physical activity in MS, and had clinical implications for patients and caregivers.

The promotion of an active lifestyle has been suggested in the treatment of a broad range of diseases that are tightly linked to metabolic and immune-mediated disarrangement, including autoimmune diseases such as MS (22, 23). Though most epidemiological studies have supported the protective role of physical activity in the risk of MS (3, 4), contradictory results have also been reported (7, 9). Using the MR approach, our results demonstrated the protective effect of moderate physical activity against MS. Similarly, previous studies showed that aerobic exercise could improve mood and reduce fatigue in patients with MS (4, 24, 25), suggesting the beneficial role of physical activity in the MS pathogenesis. The exact mechanism of such protective mechanism is still not well understood. One possible explanation is that physical activity could function as potential immunomodulatory therapy, targeting innate signaling mechanisms to modulate MS symptoms (26). Physical activity leads to a significant elevation in T-regulatory cells, decreased immunoglobulin secretion and produces a shift in the Th1/Th2 balance, and is shown to reduce inflammatory events in patients and animal models of inflammatory diseases (27, 28). Meanwhile, physical activity could promote the release of IL-6 from muscles, which functions as a myokine and has been suggested to induce an anti-inflammatory response through IL-10 secretion and IL-1β inhibition (23). Additionally, exercise-induced systemic elevation of hormones, such as cortisol and epinephrine, inhibits the secretion of pro-inflammatory tumor necrosis factor (TNF)-α by monocytes. Furthermore, exercise might exert a protective role by affecting the modulation of immune factors and stress hormones in MS (29). These observations suggested physical activity might affect the pathogenesis of MS by modulating inflammation. Moreover, physical activity could mediate the expression of neuroactive proteins, such as neuroprotective insulin-like growth factor-I, and other neurotrophic factors like brain-derived neurotrophic factor (BDNF) which might be related to neuronal survival (30). Notably, compared with moderate physical activity, vigorous physical activity was not associated with the risk of MS based on current results. Consistent with this finding, a majority of MS patients feel high-temperature intolerance which might be related to temporary exacerbation of clinical manifestations. Vigorous physical activity might increase the core body temperature, and thus bringing harm to patients with MS. Therefore, too light or too aggressive physical activity both were not beneficial for MS. Further studies investigating physical activity in MS could pay attention to the effect of excessive levels of physical activity.

There were also some limitations worth mentioning in the current study. First, only a limited number of instrumental variables were significant in the GWAS of physical activity, which was susceptible to bias. The potential causal associations in the current study should be interpreted with caution, and further replications in well-power studies were still warranted. Second, the variance explained by the instrumental variables of the exposures such as SSOE and VPA was moderate, which limited the power to detect weaker causal associations. Replication based on summary statistics from future GWAS with a larger sample size was still necessary. Third, though physical activities with different intensities were examined, current GWAS did not differentiate between types of physical activity, like swimming and running. Considering that different types of physical activity might have different benefits and involve different parts of the body, further investigation into subtypes of physical activities might provide additional insights.

In conclusion, based on results from the genetic correlation and MR analyses, we demonstrated that moderate physical activity could causally decrease the risk of MS. These results could help better understand the role of physical activity in the pathogenesis of MS, and provided some lifestyle recommendations for individuals susceptible to MS.

## Data availability statement

The original contributions presented in the study are included in the article/**Supplementary Material**. Further inquiries can be directed to the corresponding author.

## Author contributions

(1) Research project, A. Conception, B. Organization, C. Execution. (2) Statistical Analysis, A. Design, B. Execution, C. Review and Critique. (3) Manuscript, A. Writing of the First Draft, B. Review and Critique. CL, 1A, 1C, 2A, 2B, 2C, 3A. JL, 2B, 3A, 3B. TY, 3B. YX, 3B. QJ, 3B. HS, 1B, 2C, 3B. All authors contributed to the article and approved the submitted version.

## Funding

This research was supported by the funding of the National Key Research and Development Program of China (Grant No. 2021YFC2501200), the Sichuan Science and Technology Program (Grant No. 2022ZDZX0023 and 2021YJ0415) and the National Natural Science Foundation of China (Grant No. 81901294 and 81871000).

## Conflict of interest

The authors declare that the research was conducted in the absence of any commercial or financial relationships that could be construed as a potential conflict of interest.

## Publisher’s note

All claims expressed in this article are solely those of the authors and do not necessarily represent those of their affiliated organizations, or those of the publisher, the editors and the reviewers. Any product that may be evaluated in this article, or claim that may be made by its manufacturer, is not guaranteed or endorsed by the publisher.

## Abbreviations

AccAve, overall acceleration average; BMI, body mass index; GWAS, genome-wide association study; IVW, inverse variance weighted; LD, linkage disequilibrium; MR, Mendelian randomization; MS, multiple sclerosis; MVPA, moderate-to-vigorous physical activity; SSOE, 2-3 days/week or more in doing strenuous sports or other exercises for a duration of 15-30 minutes or greater; SNP, single nucleotide polymorphism; VPA, self-reported vigorous physical activity.
