# Supplementary material for: Physical activity and risk of multiple sclerosis: A Mendelian randomization study
Source: Front Immunol. 2022 Sep 21;13:872126. doi: 10.3389/fimmu.2022.872126 (PMC9532251; doi:10.3389/fimmu.2022.872126)
Supplement: Supplementary file 1 [file DataSheet_1.pdf]

Supplementary Table 1. Summary data from all GWAS used in current study.

phenotype	abbreviation	Cases	Controls	Ethnics	Number of SNPs	PMID
overall acceleration average	AccAve	91,084	-	EUR	11,826,775	29899525
moderate-to-vigorous physical activity	MVPA	377,234	-	EUR	11,838,639	29899525
vigorous physical activity	VPA	98,060	162,995	EUR	11,834,614	29899525
strenuous sports or other exercises	SSOE	124,842	225,650	EUR	11,838,159	29899525
overall activity	-	91,105	-	EUR	7,654,310	30531941
multiple sclerosis in discovery stage	-	47,429	68,374	EUR	6,304,358	31604244
multiple sclerosis in replication stage	-	14,802	26,703	EUR	8,589,719	24076602

EUR, European; SNP, single nucleotide polymorphism; GWAS, genome-wide association study; PMID, PubMed ID; AccAve, overall acceleration average; PA, physical activity; MVPA, moderate-to-vigorous PA; VPA, vigorous PA; SSOE, strenuous sports or other exercise.

Supplementary Table 2. Effect sizes can be detected with the power of 0.8 given the sample size, proportion of cases and variance explained by instrumental variables.

stage	exposure trait	sample size of outcome GWAS	proportion of cases in outcome GWAS	variance explained by instrumental variables	effect size
discovery	AccAve	115803	0.41	6.35E-04	-0.693
	MVPA	115803	0.41	1.62E-03	-0.431
	VPA	115803	0.41	6.72E-04	-0.673
	SSOE	115803	0.41	1.38E-03	-0.465
	overall activity	115803	0.41	2.00E-03	-0.386
replication	AccAve	41505	0.36	7.16E-04	-1.238
	MVPA	41505	0.36	1.63E-03	-0.787
	VPA	41505	0.36	6.72E-04	-1.291
	SSOE	41505	0.36	1.39E-03	-0.856
	overall activity	41505	0.36	1.96E-03	-0.703

GWAS, genome wide association study; AccAve, overall acceleration average; PA, physical activity; MVPA, moderate-to-vigorous PA; VPA, vigorous PA; SSOE, strenuous sports or other exercises.

Supplementary Table 3. Summary statistics of the instrumental variables used in the Mendelian randomization analysis.

exposure trait	SNP	F statistics	Exposure			Outcome		
			beta	SE	P value	beta	SE	P value
VPA	rs6667222	33.10	8.73E-03	1.52E-03	8.70E-09	2.33E-02	1.95E-02	0.23
	rs1248860	55.26	-9.77E-03	1.31E-03	1.10E-13	1.18E-02	1.62E-02	0.47
	rs2764261	45.00	9.13E-03	1.36E-03	2.00E-11	6.42E-03	1.69E-02	0.70
	rs328902	38.48	-8.79E-03	1.42E-03	5.50E-10	1.98E-02	1.75E-02	0.26
	rs13243553	42.02	8.75E-03	1.35E-03	9.00E-11	-1.16E-02	1.67E-02	0.49
	rs3781411	39.67	1.26E-02	2.00E-03	3.00E-10	2.70E-03	2.43E-02	0.91
MVPA	rs1008833	29.76	-1.71E-02	3.14E-03	4.90E-08	4.94E-02	2.31E-02	0.03
	rs1974771	33.65	-2.13E-02	3.68E-03	6.60E-09	3.59E-03	2.59E-02	0.89
	rs2114286	30.50	-1.22E-02	2.22E-03	3.30E-08	1.10E-03	1.81E-02	0.95
	rs877483	30.13	1.22E-02	2.23E-03	4.00E-08	-2.88E-02	1.68E-02	0.09
	rs2035562	34.69	-1.39E-02	2.36E-03	3.90E-09	6.58E-03	1.73E-02	0.70
	rs1972763	30.53	1.28E-02	2.32E-03	3.30E-08	-3.15E-02	1.72E-02	0.07
	rs7731915	30.02	-1.78E-02	3.25E-03	4.30E-08	1.78E-02	2.37E-02	0.45
	rs1186721	29.98	-1.30E-02	2.37E-03	4.40E-08	1.76E-02	1.77E-02	0.32
	rs921915	38.44	-1.39E-02	2.24E-03	5.70E-10	4.30E-02	1.65E-02	0.01
	rs1043595	34.48	1.44E-02	2.45E-03	4.30E-09	-1.74E-02	1.90E-02	0.36
	rs7804463	45.99	1.50E-02	2.21E-03	1.20E-11	-1.93E-02	1.63E-02	0.24
	rs2988004	34.58	-1.32E-02	2.24E-03	4.10E-09	1.83E-02	1.70E-02	0.28
	rs7326482	31.92	-1.30E-02	2.29E-03	1.60E-08	1.23E-02	1.67E-02	0.46
	rs10145335	30.88	-1.41E-02	2.54E-03	2.70E-08	8.76E-03	1.98E-02	0.66
	rs4886868	30.40	-1.25E-02	2.27E-03	3.50E-08	-2.59E-02	1.94E-02	0.18
	rs12912808	31.85	1.75E-02	3.11E-03	1.70E-08	-4.12E-02	2.54E-02	0.11
	rs429358	51.82	-2.20E-02	3.05E-03	6.10E-13	7.45E-02	2.48E-02	0.003
	rs1921981	30.22	1.30E-02	2.37E-03	3.80E-08	1.86E-02	1.80E-02	0.30
AccAve	rs34517439	29.97	3.08E-01	5.62E-02	4.40E-08	-1.16E-01	6.45E-02	0.07
	rs6775319	30.43	2.25E-01	4.08E-02	3.50E-08	-9.85E-03	1.82E-02	0.59
	rs17551090	31.34	3.32E-01	5.93E-02	2.20E-08	2.99E-02	2.64E-02	0.26
	rs12522261	30.21	2.11E-01	3.83E-02	3.90E-08	-4.71E-03	1.71E-02	0.78
	rs11012732	34.04	2.25E-01	3.86E-02	5.40E-09	-3.41E-02	1.76E-02	0.05
	rs56194509	47.68	-3.03E-01	4.39E-02	5.00E-12	5.27E-02	2.00E-02	0.01
	rs59499656	35.60	-2.28E-01	3.83E-02	2.40E-09	-8.56E-03	1.71E-02	0.62
SSOE	rs1200154	30.21	-6.29E-03	1.14E-03	3.90E-08	1.72E-02	1.67E-02	0.30
	rs2994326	29.93	-7.96E-03	1.46E-03	4.50E-08	5.21E-03	2.30E-02	0.82
	rs288070	31.57	-1.06E-02	1.89E-03	1.90E-08	3.53E-02	2.58E-02	0.17
	rs7627864	33.37	6.55E-03	1.13E-03	7.60E-09	-8.23E-03	1.63E-02	0.61
	rs62253088	82.57	1.09E-02	1.20E-03	1.00E-19	-1.41E-02	1.74E-02	0.42
	rs4865667	32.77	6.61E-03	1.16E-03	1.00E-08	-9.55E-03	1.66E-02	0.57
	rs159544	36.76	-6.97E-03	1.15E-03	1.30E-09	-1.96E-02	1.68E-02	0.24

rs10946808	37.34	-7.71E-03	1.26E-03	9.90E-10	-1.25E-02	1.81E-02	0.49
rs1265178	30.58	7.31E-03	1.32E-03	3.20E-08	1.86E-02	2.03E-02	0.36
rs896302	31.76	7.01E-03	1.24E-03	1.70E-08	-1.37E-02	1.79E-02	0.44
rs4411372	31.46	-7.02E-03	1.25E-03	2.00E-08	-9.55E-03	1.80E-02	0.60
rs1959759	32.59	6.71E-03	1.18E-03	1.10E-08	2.23E-02	1.70E-02	0.19
rs166840	44.13	7.65E-03	1.15E-03	3.10E-11	-2.09E-02	1.76E-02	0.23
rs12973258	34.92	8.57E-03	1.45E-03	3.40E-09	5.20E-02	2.22E-02	0.02

AccAve, overall acceleration average; PA, physical activity; MVPA, moderate-to-vigorous PA; VPA, vigorous PA; SSOE, strenuous sports or other exercises; SE, standard error.

Supplementary Table 4. Phenotypes significantly associated with instrumental variables of physical activity identified by PhenoScanner.

exposure trait	SNP	associated phenotype
AccAve	rs11012732	Leg fat mass right, Leg fat percentage right, Leg fat mass left, Leg fat percentage left, Waist circumference, Body mass index, Whole body fat mass, Body fat percentage, Hip circumference, Trunk fat mass, Arm fat mass left, Arm fat mass right, Trunk fat percentage, Arm fat percentage left, Arm fat percentage right, Weight, Meningioma, Invasive ovarian cancer, Serous invasive ovarian cancer, High grade serous ovarian cancer, Arm predicted mass right, Arm fat-free mass left, Forced vital capacity, best measure, Arm predicted mass left, Sodium in urine, Arm fat-free mass right
	rs12522261	Getting up in morning
	rs17551090	Impedance of arm right, Arm predicted mass right, Arm fat-free mass right, Arm predicted mass left, Impedance of arm left, Trunk fat-free mass, Trunk predicted mass, Arm fat-free mass left
	rs34517439	Basal metabolic rate, Whole body fat-free mass, Whole body water mass, Leg predicted mass right, Leg fat-free mass left, Leg fat-free mass right, Leg predicted mass left, Weight, Trunk fat-free mass, Trunk predicted mass, Arm fat-free mass left, Arm predicted mass left, Arm fat-free mass right, Arm predicted mass right, Height, Hip circumference, Arm fat mass left, Trunk fat mass, Arm fat mass right, Whole body fat mass, Leg fat mass left, Leg fat mass right, Comparative height size at age 10, Sitting height, Body mass index, Waist circumference, Impedance of leg right, Impedance of leg left, Impedance of whole body, Trunk fat percentage, Arm fat percentage left, Arm fat percentage right, Body fat percentage, Leg fat percentage left, Creatinine in urine, Lung cancer, Impedance of arm right, Impedance of arm left, Leg fat percentage right, Forced vital capacity, Forced vital capacity, best measure, Psoriasis, Forced expiratory volume in 1-second, predicted, Lung adenocarcinoma
	rs56194509	Hair or balding pattern: pattern 4, Nap during day, Forced vital capacity, Forced vital capacity, best measure, Forced expiratory volume in 1-second, Red blood cell count, Sitting height, Relative age of first facial hair, Forced expiratory volume in 1-second, best measure, Red cell distribution width, Sensitivity or hurt feelings, Hematocrit, Hemoglobin concentration, Neuroticism score, Hair or balding pattern: pattern 3, Eosinophil percentage of granulocytes, Neutrophil percentage of granulocytes, Eosinophil percentage of white cells, Forced expiratory volume in 1-second, predicted percentage, Mood swings, Pain type experienced in last month: headache, Medication for pain relief, constipation, heartburn: paracetamol, Treatment with paracetamol, Reticulocyte count, Impedance of arm left, Impedance of whole body, Eosinophil count, Peak expiratory flow, Relative age voice broke, High light scatter reticulocyte count, Hand grip strength left, Invasive ovarian cancer, Frequency of tenseness or restlessness in last 2 weeks, Sum eosinophil basophil counts, Fed-up feelings, Impedance of arm right, Qualifications: college or university degree, High grade serous ovarian cancer, Neutrophil percentage of white cells, Mouth or teeth dental problems: mouth ulcers, Serous invasive ovarian cancer, Hand grip strength right, Nervous feelings, Alcohol intake frequency, Lymphocyte percentage of white cells, Height, Irritability, Daytime dozing or sleeping, Miserableness, Heel bone mineral density, Medication for pain relief, constipation, heartburn:

		none of the above, Comparative height size at age 10, Systolic blood pressure, High light scatter percentage of red cells, Worrier or anxious feelings, Illnesses of mother: parkinsons disease, Neuroticism
	rs59499656	Trunk fat percentage, Trunk fat mass, Body fat percentage, Arm fat percentage left, Arm fat mass left, Whole body fat mass, Arm fat percentage right, Arm fat mass right, Leg fat mass right, Leg fat percentage right, Leg fat mass left, Waist circumference, Weight, Leg fat percentage left
	rs6775319	Arm fat percentage right, Body fat percentage, Arm fat percentage left, Trunk fat percentage, Leg fat percentage left
	rs148193266	n.a.
	rs9293503	Arm predicted mass right, Impedance of arm right
MVPA	rs10145335	Intelligence multi trait analysis, Duration of vigorous activity
	rs1008833	n.a.
	rs1043595	Intelligence multi trait analysis, Number of days or week of vigorous physical activity 10+ minutes
	rs1186721	n.a.
	rs12912808	n.a.
	rs1921981	Job involves heavy manual or physical work
	rs1972763	Time spent using computer, Pulse rate
	rs1974771	n.a.
	rs2035562	Arm fat-free mass right, Arm fat-free mass left, Arm predicted mass left, Trunk fat-free mass, Arm predicted mass right, Whole body water mass, Trunk predicted mass, Whole body fat-free mass, Basal metabolic rate, Impedance of arm right, Impedance of arm left, Impedance of whole body, Leg predicted mass right, Leg fat-free mass right, Weight, Leg fat-free mass left, Leg predicted mass left
	rs2114286	Sitting height, Heel bone mineral density, Height, Basal metabolic rate
	rs2942127	n.a.
	rs2988004	n.a.
	rs429358	Alzheimers disease, Illnesses of mother: alzheimers disease or dementia, Illnesses of father: alzheimers disease or dementia, Self-reported high cholesterol, Late onset Alzheimers disease, Treatment with cholesterol lowering medication, Dementia with Lewy bodies, Medication for cholesterol, blood pressure or diabetes: cholesterol lowering medication, Treatment with simvastatin, Advanced age related macular degeneration, Age-related macular degeneration, Treatment with atorvastatin, Cerebral amyloid deposition PET imaging, Illnesses of siblings: alzheimers disease or dementia, APOE apolipoprotein E, Parental lifespan, Coronary artery disease, Red cell distribution width, Low density lipoprotein, C-reactive protein, APOE apolipoprotein E females, Lifespan, Illnesses of mother: none of the above, group 1, Posterior cortical atrophy, Cerebral amyloid deposition positivity PET imaging, Total cholesterol, Alzheimers disease age of onset, Treatment with ezetimibe, Cerebrospinal AB1 42 levels in Alzheimers disease dementia, Treatment with lipitor 10mg tablet, Waist circumference, Medication for cholesterol, blood pressure or diabetes: none of the above, Plateletcrit, Mother still alive, Leg fat percentage left, HDL cholesterol, Leg fat mass left, Cognitive decline age

		related, Illnesses of mother: diabetes, Leg fat percentage right, Weight change compared with 1 year ago, Alzheimers disease biomarkers, Cortical amyloid beta load, Self-reported dementia or alzheimers/cognitive impairment, Leg fat mass right, Treatment with rosuvastatin, Body mass index, No treatment with medication for cholesterol, blood pressure, diabetes, or take exogenous hormones, Blood protein levels, Body fat percentage, Whole body fat mass, Cognitive ageing females, Arm fat mass right, APOE apolipoprotein E males, Lewy body disease, Arm fat mass left, Cognitive ageing, Trunk fat mass, Type II diabetes, Trunk fat percentage, LDL cholesterol change with statins, Weight, Pulse rate, Cause of death: alzheimers disease, unspecified, Arm fat percentage left, Arm fat percentage right, Illnesses of father: none of the above, group 1, Chronic ischaemic heart disease, Platelet count, Treatment with ezetrol 10mg tablet, Fathers age at death, Mothers age at death, Illnesses of father: diabetes, Cause of death: unspecified dementia, Myocardial infarction, Frequency of stair climbing in last 4 weeks
	rs4886868	Height, Comparative height size at age 10, Forced vital capacity, best measure, Sitting height, Forced vital capacity, Trunk predicted mass, Trunk fat-free mass, Hand grip strength left, Hand grip strength right
	rs7326482	Overall health rating
	rs7731915	n.a.
	rs77742115	n.a.
	rs7804463	Number of days or week of vigorous physical activity 10+ minutes
	rs877483	n.a.
	rs921915	Monocyte percentage of white cells, Monocyte count
VPA	rs1248860	Trunk fat-free mass, Trunk predicted mass, Types of physical activity in last 4 weeks: other exercises, Arm predicted mass left, Comparative body size at age 10, Whole body water mass, Whole body fat-free mass, Arm fat-free mass left, Frequency of tiredness or lethargy in last 2 weeks, Arm fat-free mass right, Arm predicted mass right, Impedance of arm left, Past tobacco smoking, Impedance of whole body
	rs13243553	Number of days or week of vigorous physical activity 10+ minutes, Intelligence, Number of days or week of moderate physical activity 10+ minutes
	rs2764261	Basal metabolic rate, Whole body water mass, Whole body fat-free mass, Trunk predicted mass, Trunk fat-free mass, Arm fat-free mass left, Arm predicted mass left, Arm fat-free mass right, Arm predicted mass right, Weight, Leg fat-free mass left, Leg predicted mass left, Leg fat-free mass right, Leg predicted mass right, Height, Sitting height, Whole body fat mass, Leg fat mass left, Waist circumference, Trunk fat mass, Leg fat mass right, Hip circumference, Arm fat mass right, Arm fat mass left, Comparative height size at age 10, Body mass index, Impedance of whole body, Impedance of arm left, Age at menarche, Impedance of arm right, Intelligence, Mean time to correctly identify matches, Fluid intelligence score, Leg fat percentage left, Body fat percentage, Trunk fat percentage, Leg fat percentage right
	rs328902	n.a.
	rs3781411	n.a.
	rs6667222	Number of days or week of vigorous physical activity 10+ minutes
SSOE	rs10946808	Height, Mean corpuscular hemoglobin, Trunk fat mass, Weight, Sitting height, Trunk fat percentage, Whole body fat mass, Comparative height size at age 10, Mean corpuscular volume,

	Hip circumference, Basal metabolic rate, Leg fat-free mass left, Leg predicted mass left, Body fat percentage, Leg fat mass left, Leg fat mass right, Whole body fat-free mass, Hip circumference adjusted for BMI, Arm fat-free mass right, Whole body water mass, Arm fat-free mass left, Arm predicted mass left, Leg predicted mass right, Leg fat-free mass right, Arm predicted mass right, Trunk fat-free mass, Trunk predicted mass, Arm fat mass left, Arm fat mass right, Mouth or teeth dental problems: dentures, Leg fat percentage left, Mean corpuscular hemoglobin concentration, Red cell distribution width, Leg fat percentage right, Height in females, Waist circumference, Body height, Qualifications: college or university degree, Arm fat percentage left, Arm fat percentage right, Waist circumference adjusted for BMI, Time spent watching television, Forced expiratory volume in 1-second, predicted, Height tails, Height in males, Weight in females, Pack years of smoking preview only, Waist circumference adjusted for physical activity, Disorders of mineral metabolism, Vascular or heart problems diagnosed by doctor: none of the above, Waist circumference adjusted for smoking, Qualifications: A levels or as levels or equivalent, Hip circumference in males, Hayfever, allergic rhinitis or eczema, Pack years adult smoking as proportion of life span exposed to smoking, Height females, Vascular or heart problems diagnosed by doctor: high blood pressure, Waist circumference in physically active indivdiuals, Birth weight, Self-reported hypertension, Waist circumference in non-smokers, Waist circumeference adjusted for physical activity in males, Body mass index
rs1200154	n.a.
rs1265178	n.a.
rs12973258	Height, Low density lipoprotein, Total cholesterol, Triglycerides, Comparative height size at age 10, Leg fat percentage left, Leg fat percentage right, Sitting height, Age at first live birth, Impedance of arm right, Red cell distribution width
rs159544	Qualifications: college or university degree, Qualifications: none, Schizophrenia, Years of educational attainment, Trunk fat-free mass, Trunk predicted mass
rs166840	Mean corpuscular hemoglobin, Mean corpuscular volume, Mean platelet volume, Types of physical activity in last 4 weeks: other exercises, Platelet count
rs1959759	Frequency of tiredness or lethargy in last 2 weeks, Overall health rating
rs288070	n.a.
rs2994326	n.a.
rs4411372	Leg fat percentage right, Body fat percentage, Trunk fat percentage, Arm fat percentage left, Arm fat percentage right, Whole body fat mass, Leg fat mass right, Trunk fat mass
rs4865667	Heel bone mineral density
rs62253088	Types of physical activity in last 4 weeks: other exercises, Comparative body size at age 10, Suffer from nerves, Impedance of arm left, Impedance of arm right, Nervous feelings, Impedance of whole body, Arm fat-free mass right, Trunk fat-free mass, Trunk predicted mass, Arm predicted mass left
rs7627864	Qualifications: college or university degree, Time spent watching television, Body mass index, Years of educational attainment, Leg fat mass right, Leg fat mass left, Weight, Arm fat mass right, Arm fat mass left, Impedance of arm right, Impedance of arm left, Whole body fat mass, High light scatter reticulocyte count, Arm predicted mass left, High light scatter percentage of red cells, Qualifications: A levels or as levels or equivalent, Leg fat percentage right, Arm fat-

		free mass left, Arm predicted mass right, Leg fat percentage left, Impedance of whole body, Basal metabolic rate, Years of educational attainment in females, Arm fat-free mass right, Reticulocyte count, Reticulocyte fraction of red cells, Trunk fat mass, Qualifications: none, Waist circumference, Arm fat percentage right, Whole body water mass, Leg predicted mass left, Whole body fat-free mass, Arm fat percentage left, Leg fat-free mass left, Job involves heavy manual or physical work, Body fat percentage, Overall health rating, Immature fraction of reticulocytes, Age at first live birth, Years of educational attainment in males, Trunk fat-free mass, Hip circumference, Trunk predicted mass, Age at menarche, Leg fat-free mass right, Age completed full time education, Leg predicted mass right, Age at last live birth, Age first birth, Inflammatory bowel disease, Trunk fat percentage, Types of physical activity in last 4 weeks: other exercises, Average weekly beer plus cider intake, Taking other prescription medications, Number of treatments or medications taken, Impedance of leg left, Miserableness
	rs896302	n.a.
	rs75930676	n.a.
Overall activity	rs11012732	Leg fat mass right, Leg fat percentage right, Leg fat mass left, Leg fat percentage left, Waist circumference, Body mass index, Whole body fat mass, Body fat percentage, Hip circumference, Trunk fat mass, Arm fat mass left, Arm fat mass right, Trunk fat percentage, Arm fat percentage left, Arm fat percentage right, Weight, Meningioma, Invasive ovarian cancer, Serous invasive ovarian cancer, High grade serous ovarian cancer, Arm predicted mass right, Arm fat-free mass left, Forced vital capacity, best measure, Arm predicted mass left, Sodium in urine
	rs12522261	Getting up in morning
	rs59499656	Trunk fat percentage, Trunk fat mass, Body fat percentage, Arm fat percentage left, Arm fat mass left, Whole body fat mass, Arm fat percentage right, Arm fat mass right, Leg fat mass right, Leg fat percentage right, Leg fat mass left, Waist circumference, Weight, Leg fat percentage left
	rs62055696	Red blood cell count, Red cell distribution width, Hematocrit, Hemoglobin concentration, Eosinophil percentage of granulocytes, Neutrophil percentage of granulocytes, Eosinophil percentage of white cells, Eosinophil count, Reticulocyte count, High light scatter reticulocyte count, Sum eosinophil basophil counts, Neutrophil percentage of white cells, Invasive ovarian cancer, Lymphocyte percentage of white cells, High grade serous ovarian cancer, Serous invasive ovarian cancer, Neuroticism, Mean platelet volume, High light scatter percentage of red cells, Sum basophil neutrophil counts, Neutrophil count
	rs6775319	Arm fat percentage right, Body fat percentage, Arm fat percentage left, Trunk fat percentage, Leg fat percentage left
	rs6895232	n.a.

n.a., not available; AccAve, overall acceleration average; PA, physical activity; MVPA, moderate-to-vigorous PA; VPA, vigorous PA; SSOE, strenuous sports or other exercises.
