# Supplementary material for: Physical activity and risk of multiple sclerosis: A Mendelian randomization study
Source: Front Immunol. 2022 Sep 21;13:872126. doi: 10.3389/fimmu.2022.872126 (PMC9532251; doi:10.3389/fimmu.2022.872126)

Supplementary Figure 1. Assumptions in Mendelian randomization analysis.

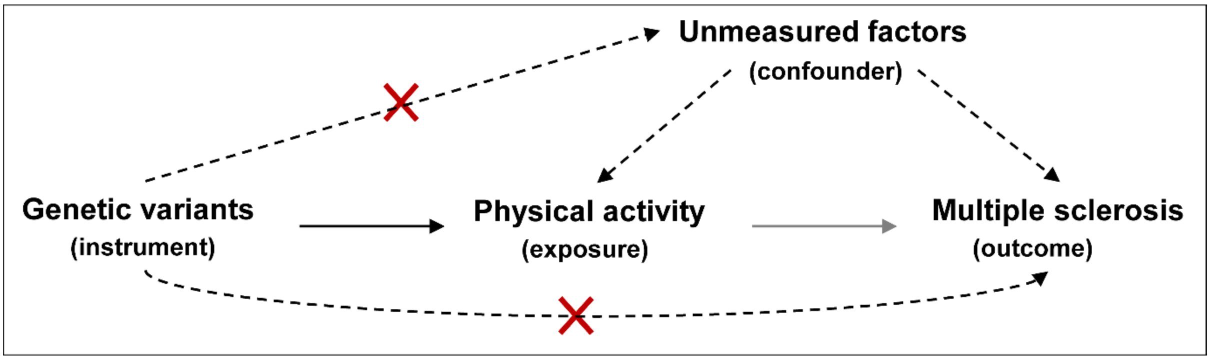

Supplementary Figure 2. Mendelian randomization analysis results for AccAve on risk of multiple sclerosis.

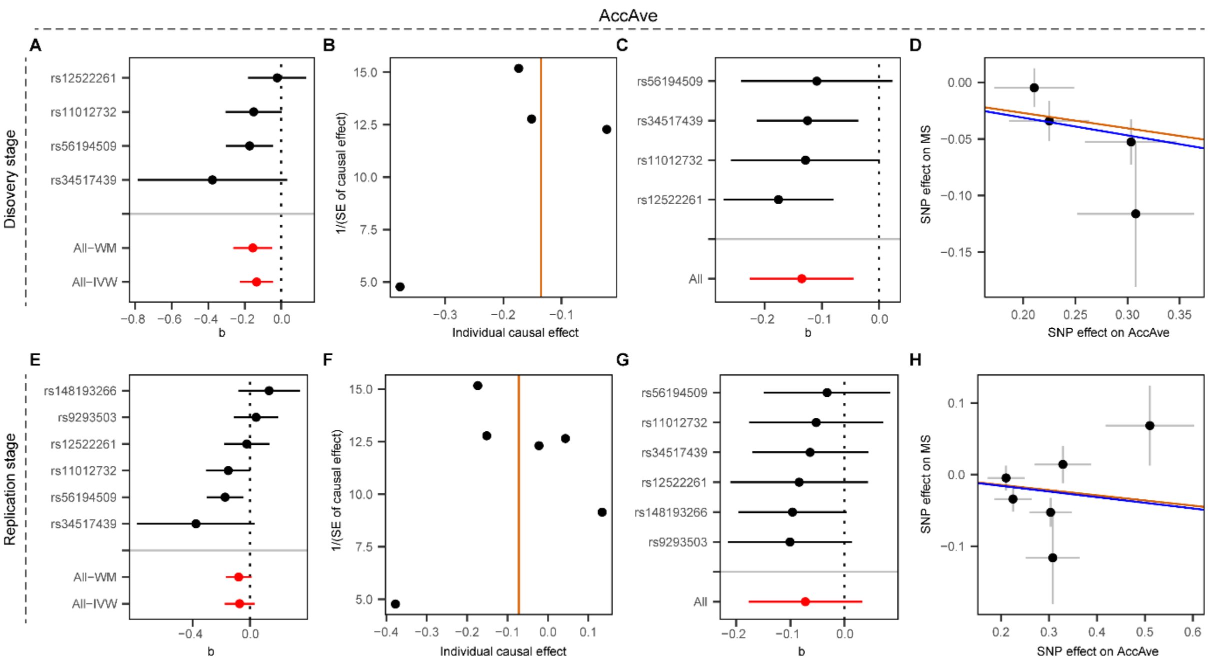

(**A,E**) Forest plot of the causal effect of AccAve on multiple sclerosis (MS). AccAve, overall acceleration average. (**B,F**) Funnel plot showing the estimation using the inverse of the standard error of the causal estimate with each individual single nucleotide polymorphism (SNP) as a tool. The vertical line represents the estimated causal effect. (**C,G**) Forest plot of the results of the leave-one-out sensitivity analysis, where each SNP was iteratively removed from the instrument variables. (**D,H**) Scatter plot of SNP potential effects on AccAve and MS. The 95% CI for the effect size on multiple sclerosis is shown as vertical lines, while the 95% CI for the effect size on AccAve is shown as horizontal lines. The slope of fitted lines represents the estimated MR effect.

Supplementary Figure 3. Mendelian randomization analysis results for MVPA on risk of multiple sclerosis.

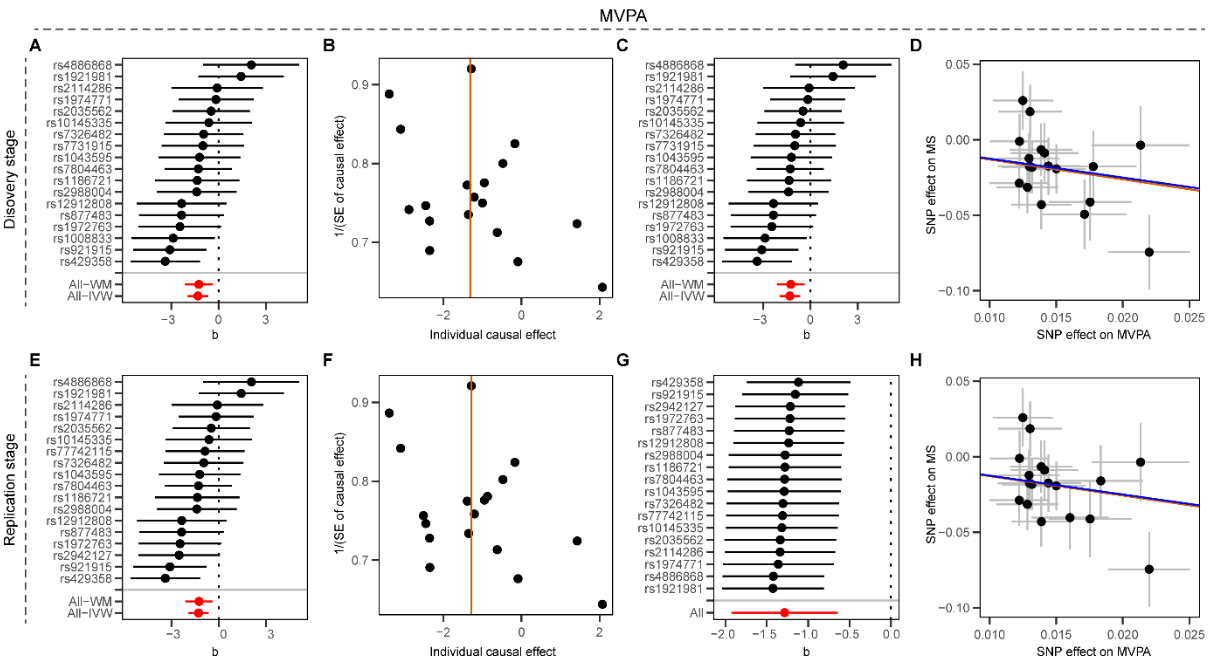

MVPA, moderate-to-vigorous physical activity.

Supplementary Figure 4. Mendelian randomization analysis results for SSOE on risk of multiple sclerosis.

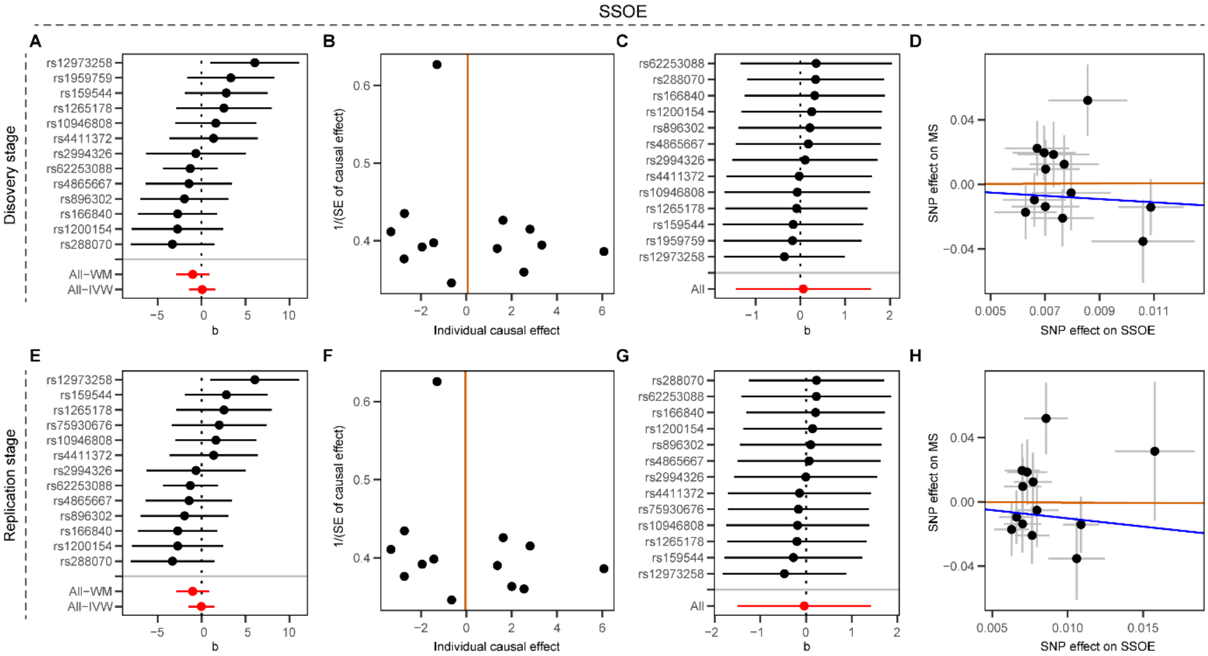

SSOE, strenuous sports or other exercises.

Supplementary Figure 5. Mendelian randomization analysis results for VPA on risk of multiple sclerosis.

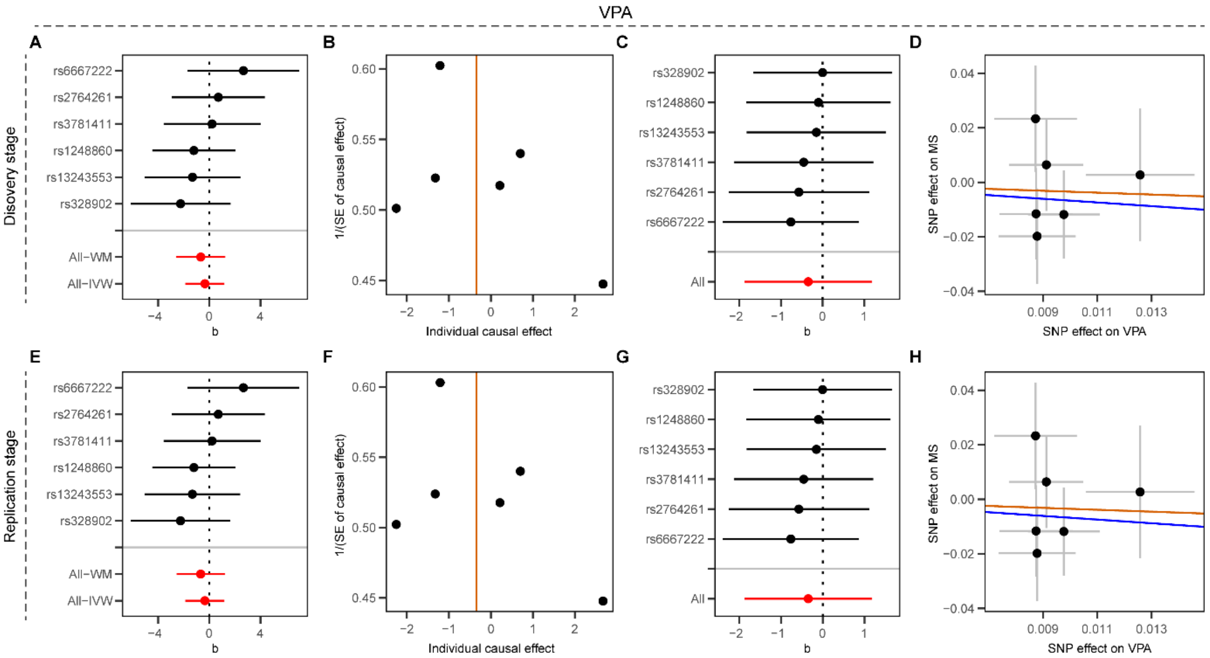

VPA, vigorous physical activity.

Supplementary Figure 6. Mendelian randomization analysis results for overall activity on risk of multiple sclerosis.

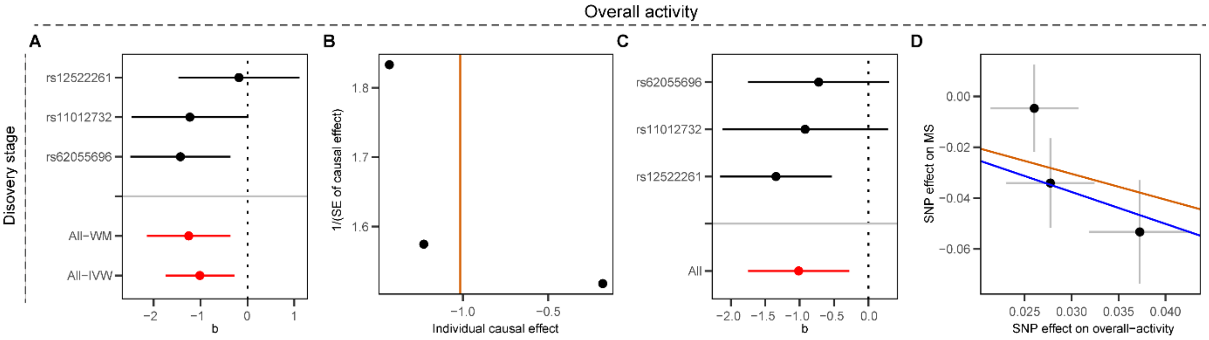

Supplement: Supplementary file 2 [file DataSheet_2.pdf]
